# Efficient Degradation of Consumer-Grade PLA by Commercial Savinase: Optimized Conditions and Molecular Dynamics Insights

**DOI:** 10.1021/acssuschemeng.5c03378

**Published:** 2025-06-09

**Authors:** Marija Nenadović, Marijana Ponjavić, Brana Pantelic, Maciej Guzik, Tomasz M. Majka, Georgia Sourkouni, Aleksandra Maršavelski, Jasmina Nikodinovic-Runic

**Affiliations:** † Institute of Molecular Genetics and Genetic Engineering, 54801 University of Belgrade, Vojvode Stepe 444a, Belgrade 11000, Serbia; ‡ Jerzy Haber Institute of Catalysis and Surface Chemistry, 132074 Polish Academy of Sciences, Niezapominajek 8, Krakow 30-239, Poland; § Department of Chemistry and Technology of Polymers, Faculty of Chemical Engineering and Technology, 49571 Cracow University of Technology, Warszawska 24, Krakow 31-155, Poland; ∥ Clausthal Centre for Materials Technology (CZM), 536335 Clausthal University of Technology, Leibnizstraße 9, Clausthal-Zellerfeld 38678, Germany; ⊥ Faculty of Science, University of Zagreb, Horvatovac 102a, Zagreb 10000, Croatia

**Keywords:** poly(lactic acid), polylactide, bioplastics, Savinase, enzymatic degradation, biodegradation, biocatalysis

## Abstract

Poly­(lactic acid) (PLA) is the most widely produced biopolymer globally, with 920,000 tons reaching the market in 2024. Although it is a biobased polymer and biodegradable, it currently poses a threat by generating a considerable waste stream in the environment unless its end-of-life options are further developed. Biorecycling of PLA is a promising solution and is critical for PLA to be a truly sustainable alternative to conventional plastics, further enabling the (bio)­plastics circular economy. This work investigates the repurposing of commercial Savinase 12T preparation, already produced on a large scale for the detergent industry, for the degradation of consumer-grade PLA. Savinase-degraded postconsumer PLA single-use cups at a rate of 166 mg·day^–1^·mg enzyme^–1^ for the lid part (crystallinity (*X*_c_) 22.9%) and 40 mg·day^–1^·mg enzyme^–1^ for the body part (*X*_c_ 42.0%). The average degradation rate of PLA 3D printer filaments was determined to be 56 mg·day^–1^·mg enzyme^–1^ under optimized conditions of pH 8.5 and 42 °C. Lactic acid was identified as the main PLA degradation product of Savinase depolymerizing activity. Molecular modeling revealed Asn155 and Ser125 as key residues involved in PLA tetramer binding.

## Graphical abstract

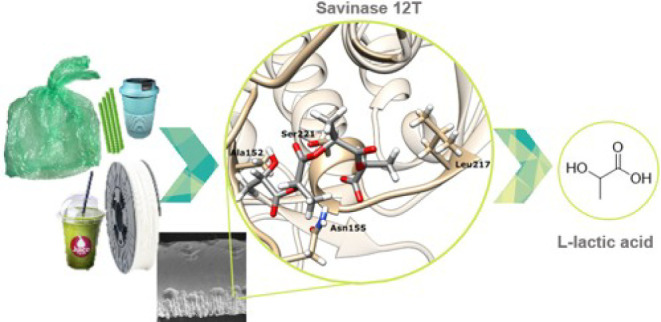

## Introduction

Poly­(lactic acid) (PLA) is a polyester industrially produced through chemical polymerization of lactic acid, which can be obtained by microbial fermentation of renewable biomass.^1^ Commercial-grade PLA consists primarily of L-lactic acid (>95%) with a minor fraction of D-lactic acid (<5%),^2^ along with the addition of usually undisclosed additives.^3^ The mechanical, thermal, optical, and barrier properties of PLA are similar to those of poly­(ethylene terephthalate) (PET) and poly­(styrene) (PS),^4^ making it a suitable biobased alternative for 11.5% of the fossil-based plastics produced.^5^ Its mechanical properties enable processing with existing technologies such as injection molding and extrusion.^1^ With production costs under 2 $/kg, comparable to PS, PLA dominates the bioplastic market with 24% of production volume.^6^ It is predominantly used in single-use products and packaging, contributing to ∼40% of global plastic waste, with a market size of ∼143 million metric tons in 2021.^5,7^ Additionally, PLA is increasingly used in 3D printing across aerospace, military, medical, and construction industries.^8^ This trend is expected to increase PLA waste, as discarded prototypes and failed prints contribute to waste.

In addition to favorable mechanical properties, PLA’s marketing as a biodegradable alternative to conventional plastics fostered its success as a novel polymer, although it does not degrade in open environments.^9^ PLA can biodegrade under controlled conditions in industrial composting facilities at >58 °C and high humidity, with the degradation rate varying from 65% to 80% within 80 to 180 days.^10^ However, composting has its shortcomings, including the release of PLA microplastics and the loss of monomers, making it unsuitable for a circular PLA economy.^11^

In contrast, enzymatic depolymerization of PLA produces optically pure lactic acid monomers at mild temperatures.^12^ Moreover, the high enzyme specificity allows for PLA degradation in mixed plastic waste, mitigating the plastic sorting problem, which is particularly important since PLA severely impairs the recycling of petrochemical plastics and vice versa.^13^ Recovered monomers from existing PLA waste streams can be continually repolymerized to produce virgin-grade PLA^6,12^ or upcycled into bacterial nanocellulose^14^ or ionic liquids.^15^

Enzymatic PLA degradation was first reported in the 1980s with Proteinase K (EC 3.4.21.64),^16^ and this enzyme remains the benchmark enzyme in PLA degradation studies to date. Since then, many PLA depolymerases have been characterized and classified into PLA depolymerase type I (proteases, EC 3.4.-.-) and type II (esterases, lipases, cutinases, EC 3.1.1.-), which are specific toward PLA consisting of L-lactic acid and D-lactic acid, respectively.^17^ Proteases are particularly significant as they are specific for poly­(L-lactic acid), which constitutes the majority of PLA on the market.^2,17^ In 2000, Oda et al. reported that among 56 commercial protease preparations, Proteinase K was the best-performing enzyme for PLA depolymerization, followed by Savinase (EC 3.4.21.62), which achieved 50% of Proteinase K’s efficiency.^18^ However, testing was performed at 50 °C, the optimal conditions for Proteinase K.^19^ On the other hand, Savinase is a significant enzyme due to its established large-scale production and immobilization pipelines for application in the detergent industry at temperatures lower than 40 °C.^20^ Therefore, in this study, immobilized Savinase 12 T was further explored with regard to its PLA depolymerizing activity. Reaction conditions were optimized, and a wide scope of postconsumer PLA items, in contrast to the usually employed PLA powders/films with low PLA crystallinity, were assayed as substrates to determine its applicability for biocatalytic (re)­cycling as a sustainable end-of-life option for PLA items.

## Experimental Section

### Materials

All chemicals and substrates used in this study were purchased from Merck (Munich, Germany), unless specified otherwise. Different grades of PLA polymers were obtained from suppliers listed in Table S1. Poly­(hydroxybutyrate) (PHB) was obtained from Biomer (Schwalbach, Germany), and poly­(hydroxyoctanoate) (PHO) was sourced from Bioplastech (Dublin, Ireland). PLA (CAS No. 26100-51-6, Mw = 116 kDa, PDI = 1.83, dedicated for injection molding), with the trade name Ingeo Biopolymer 3052D, was purchased from NatureWorks (Minneapolis, USA). Poly­(ε-caprolactone) (PCL) (viscosity of 0.40 dL/g) was also purchased from Merck (Munich, Germany).

Savinase 12T (batch GHSFS-1-02-1) and Alcalase 2.4 L FG (batch PLN05554) were kindly provided by Novozymes (Copenhagen, Denmark). The immobilized Savinase 12T preparation is reported to contain 1% (w/w) of Savinase enzyme.^21^ Proteinase K from Tritirachium album (batch 230006) was obtained from Serva (Heidelberg, Germany). Lipase immobilized on Immobead 150 from Rhizomucor miehei (batch 52001) and lipase immobilized on Immobead 150 from Pseudomonas cepacia (batch 54327) were purchased from Merck (Munich, Germany), and lipase for the dairy industry was supplied by Serowar PL (Szczecin, Poland).

### Esterase Activity of Savinase and Proteinase K with Monoester Substrate

Esterase activity of Savinase and Proteinase K preparations was measured in a 96-well plate at 25 °C. The reaction was initiated by the addition of 25 μL of 0.1 mg·mL^–1^ enzyme into 100 μL of 1 mM pNP-butyrate in 0.1 M Tris buffer, pH 8.5. Samples containing inactivated enzyme, prepared by incubation at 80 °C for 30 min, were included for each enzyme. Spontaneous aqueous substrate hydrolysis during the reaction course was quantified in samples containing no enzyme preparation. Each reaction was run in triplicate. The concentration of pNP was calculated using the molar extinction coefficient ε = 18,000 M^–1^ cm^–1^,^22^ and specific esterase activity was determined for commercial preparations of Savinase and Proteinase K. One unit of esterase activity (1U) is defined as 1 μmol of *p*-nitrophenol released by the enzyme preparation per minute under assay conditions. Specific esterase activities were expressed as U/mg enzyme.

### Depolymerization of Biopolyesters in Agarose Plates

Polyesterase activity of Savinase was tested against PLA, PHB, PHO, and PCL using the clearing-zone method on agarose plates with suspended biopolyesters. Polymer plates were prepared using 0.1 M Tris buffer (pH 8.5), as previously described.^22^ The perforated well in the polymer plate was loaded with 40 μL of 3.3 mg·mL^–1^ Savinase 12T solution in 0.1 M Tris buffer (pH 8.5). Polymer plates were incubated at 42 °C for 10 days and monitored for the appearance of halos around the well.

### PLA Sample Preparation and Characterization

The list of PLA samples, along with their suppliers, dimensions, and initial weights of all tested specimens, is provided in Table S1. PLA Ingeo 4043D from NatureWorks (Minneapolis, USA) was used to prepare films (a-Film) of 0.35 mm thickness as previously described.^14^ hc-Film, with a thickness of 1 mm, was sourced from Pardam (Roudnice, Czech Republic). Extruded PLA fiber (e-Filament) was prepared from a biopolymer dedicated to injection molding (Ingeo 3052D, NatureWorks, Mw = 116 kDa, PDI = 1.83) under the processing conditions shown in Table S1. Using the same extrusion processing line, PLA/Savinase 12T composites containing 0.5, 1.0, and 1.5 wt % of enzyme were obtained following the procedure described in Supporting Information. Different parts of a single-use, PLA cup were cut into 1 cm × 1 cm films for testing, with these samples denoted as b-Cup for the bottom part and l-Cup for the lid. A PLA-based durable, reusable coffee cup was denoted as t-Cup and had an approximate thickness of 2.45 mm. PLA filaments for 3D printing, with a fixed radius, were cut into 1 cm pieces (3DP-Filament). PLA samples, both before and after degradation, were analyzed by confocal laser scanning microscopy (CLSM), Fourier transform infrared spectroscopy (FTIR), differential scanning calorimetry (DSC), and field emission scanning electron microscopy (FESEM), as described in Supporting Information.

### Enzymatic PLA Degradation

Enzyme mix stock solution containing 5 mg·mL^–1^ Savinase 12T, 5 mg·mL^–1^ Alcalase, and 1.5 mg·mL^–1^ of each lipase (Lipase Serowar, lipase from R.miehei and lipase from P. cepacia) in 0.1 M Tris buffer at pH 8.5, supplemented with 0.05% w/v sodium azide as an inhibitor of microbial growth, was used for the degradation experiments.^23^ Individual enzyme stock solutions were also prepared in the same buffer at the same concentrations as in the enzyme mix. The prepared enzyme stock solutions were stored in 1 mL aliquots at −20 °C for no longer than 4 weeks.

PLA degradation reactions were initiated by the addition of 1 mL of enzyme stock solution into a glass vial containing 4 mL of buffer and a PLA sample. Every reaction was performed in duplicate. The initial mass of each PLA sample is provided in Table S1. Samples containing inactivated enzymes and samples with no enzyme preparations were also included. Reactions were incubated at 42 °C and 220 rpm. Every week, 1 mL of the reaction mixture was exchanged with 1 mL of enzyme stock solution. To measure PLA weight loss, PLA specimens were washed three times with water, wiped with 70% ethanol, and air-dried. The described methodology was employed over the course of 4 weeks. To determine the optimum conditions for PLA depolymerization by Savinase, reactions were incubated at 30 °C, 35 °C, 40 °C, 42 °C, 47 °C, and 50 °C, with pH values of 5.5, 7, 8.5, 10, and 11. Buffers used for pH optimum determination included sodium acetate (pH 5.5), sodium phosphate (pH 7), Tris (pH 8.5), and sodium bicarbonate (pH 10 and 11), all at 0.1 M concentrations. For assaying the degradation of consumer-grade PLA items over 2 weeks, the same reaction setup was employed, with the addition of degradation medium exchange every 2 days to ensure both pH control and constant enzyme load. Weight loss was determined and expressed as wt %. Reactions were stopped when the samples degraded completely. Reaction aliquots (1 mL) taken after 1.5, 3, and 24 h were kept at −20 °C until HPLC and LC-MS analysis.

### PLA Degradation in Model Compost Supplemented with Savinase

Degradation of commercially available PLA bags was investigated in model compost supplemented with Savinase 12T. PLA bags were cut into rectangular 10 cm × 5 cm pieces. The compost consisted of an 80:20 (w/w) mixture of gardening soil and mature compost (ACS Garden, Belgrade, Serbia). The mixture was dried at 60 °C for 24 h before being adjusted to 50% water content using sterile water, consistent with the ASTM D5338-15 standard. 2 kg of the prepared compost was placed into two containers, one serving as the control and the other supplemented with 10 g/kg of Savinase 12T. PLA bag pieces were buried in both setups and incubated at 30 °C for six months, with weekly additions of 30 mL of sterile water to maintain moisture. At the end of the incubation period, the bags were retrieved, inspected visually, cleaned, dried at 60 °C for 16 h, analyzed using FTIR and DSC, and weighed to evaluate the mass loss and the extent of degradation.^24^

### HPLC and LC-MS Assessment of Enzymatic Depolymerization Products

PLA hydrolysate aliquots (1 mL) obtained during Savinase and Proteinase K PLA degradation were prepared for HPLC and LC-MS analysis by the addition of 6 M HCl (50 μL), vortexing, and centrifuging at 12,000 × rpm for 10 min (Eppendorf Centrifuge 5417 R). The obtained supernatants were filtered through 0.2 μm syringe filters into vials. HPLC and LC-MS run conditions are provided in Supporting Information. Lactic acid concentration in the samples was quantified using the lactic acid standard calibration curve (*y* = 11.26*x* + 0.0506, *R* ^2^ = 0.9999). The relative abundance of each HPLC peak was calculated as the percentage of the individual peak area relative to the total peak area. L-Lactic acid dimer (dimer) was detected in negative mode at *m*/*z* 161, along with its corresponding fragments at *m*/*z* 89 and 45 (Figure S1). Other PLA oligomers were not detected under the described conditions.

### Molecular Dynamics Simulations

The parameterization of Savinase^25^ and its associated dimer and tetramer of the L-lactate ligand for AMBER molecular dynamics (MD) simulations was conducted using the AMBER ff19SB force field^26^ for the protein and the General AMBER Force Field (GAFF) for the ligand.^27^ The initial structure of the PLA dimer and tetramer was prepared using Antechamber, where partial atomic charges were assigned via the AM1-BCC method. Missing force field parameters for the dimer and tetramer of L-lactate were generated using parmchk2. Similarly, the ester dimers of three representative monomers, 6-hydroxyhexanoic acid (representing PCL), 3-hydroxybutyric acid (representing PHB), and 3-hydroxyoctanoic acid (representing PHO), were used. These dimers were docked into the crystal structure of Savinase. The system was solvated in an OPC water box with a 10 Å buffer and neutralized with Cl^–^ ions, and its coordinates were verified by using the check and charge commands in tleap to ensure proper parameterization. The systems underwent a four-stage minimization process to remove steric clashes and optimize the structure. Min1 involved 1,500 steps of minimization with a distance-dependent dielectric and a 10.0 Å cutoff, while restraining all protein heavy atoms with a force constant of 50 kcal/mol·Å^2^. In Min2, the restraints were reduced to backbone atoms only (C, O, N, and Ca) with the same force constant. Min3 further relaxed the system by applying weaker backbone restraints (20 kcal/mol·Å^2^), and Min4 performed an unrestrained minimization (15,000 steps) to ensure global stability. For equilibration, the system was first heated from 0 to 300 K under NVT conditions (constant volume) for 50 ps, with backbone restraints (50 kcal/mol·Å^2^) and SHAKE-enabled hydrogen bonds. The second equilibration (eq2) switched to NPT conditions for 50 ps, maintaining 300 K with a Langevin thermostat, while removing positional restraints and enabling periodic boundary conditions. Finally, the production MD run was performed under the NPT ensemble with a 10.0 Å cutoff, a 2 fs time step, and 300 K temperature control. Trajectories were saved every 1,000 steps, and energy/data were recorded every 100 steps for analysis. This protocol ensures proper system relaxation before unrestrained production dynamics. The molecular dynamics protocol was executed in three independent replicas for each complex to ensure the statistical robustness of the results. The production runs were performed using GPU-accelerated code^28,29^ from AMBER22.^30^

## Results and Discussion

Building upon our previous study,^14^ in which a five-enzyme mixture—comprising Savinase, Alcalase, Serowar PL lipase (Lipase 1), lipase from R. miehei­(Lipase 2), and lipase from P. cepacia (Lipase 3)—was successfully utilized to completely degrade PLA films over 8 and 16 weeks, we aimed to optimize the experimental parameters and evaluate the individual contributions of each enzyme in the current study. In contrast to the initial study, when the PLA film of lower crystallinity (a-Film, *X*_c_ 7.3%) was used, we have used a film of 4-fold higher crystallinity (hc-Film, *X*_c_ 29.6%) to assess the individual enzymes for their efficiency in PLA degradation (Figure 1A). Apart from Savinase, the individual enzymes did not achieve significant PLA depolymerization, even at different temperatures of up to 50 °C (data not shown). Savinase achieved 2.25-fold higher PLA degradation with hc-Film in comparison to the enzyme mix and outperformed every other enzyme from the mix (Figure 1A). Using Savinase alone, the required enzyme load was reduced by 200-fold compared to the enzyme load in the original enzyme mixture. Overall, hc-Film degraded at a slower rate under the tested conditions in comparison to a-Film, confirming the contribution of crystallinity to the polymer degradation rate. Savinase exhibited PLA-degrading activity under a range of tested reaction conditions (pH 7–10 and 30 °C–50 °C). The optimum conditions for the PLA-degrading activity of Savinase were determined to be pH 8.5 and 42 °C (Figure 1B,C). These findings are consistent with reported values for a number of other alkaline proteases, which exhibit PLA-degrading activity at pH 8–9 and 37 °C–60 °C.^31^ Therefore, Savinase is among the PLA depolymerases active at the lower end of temperatures, which can contribute to energy savings during PLA depolymerization. PLA degradation by Savinase was further optimized through pH control, as the released lactic acid could lower the pH of the reaction to suboptimal values.

**Figure 1:**
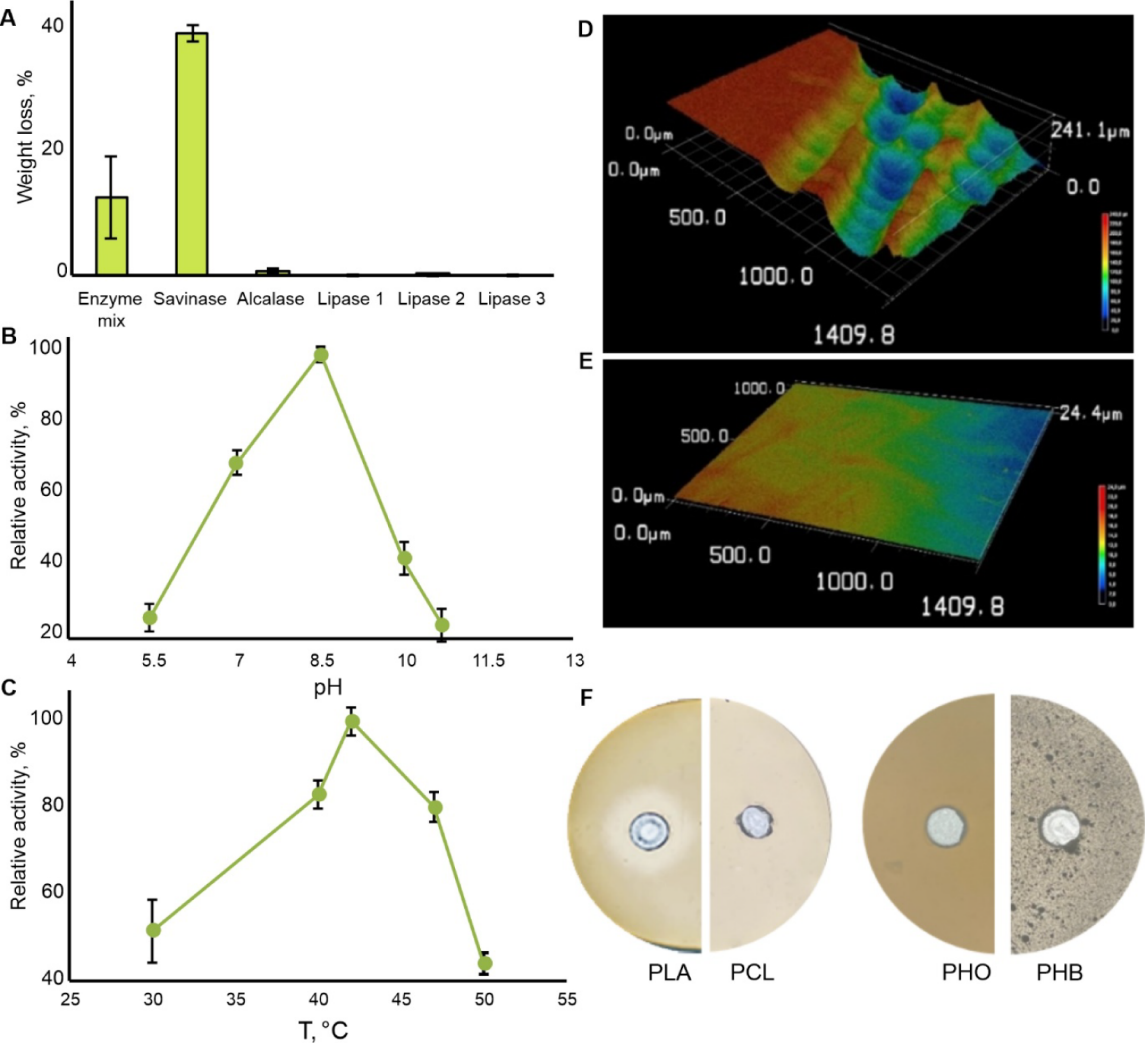
Depolymerizing activity of Savinase on PLA and other biopolymers. (A) Comparison with other enzymes and enzyme mix using PLA hc-film; (B) pH optimum for PLA degradation; (C) temperature optimum for PLA degradation; (D) 3D micrograph of PLA film incubated with Savinase; (E) 3D micrograph of PLA film incubated in buffer. (F) Degradation of biopolyesters in agar plates upon incubation at 42 °C for 10 days.

PLA film degradation by Savinase was visualized using CLSM (Figure 1D,E). Progressive disappearance of the polymer matrix, starting from the film ends and resulting in high surface roughness with erosion dips reaching up to 200 μm, was observable (Figure 1D), while the film incubated in the buffer retained its smooth appearance (Figure 1E). Although Savinase is a broad-specificity protease, it exhibits high specificity as a polyesterase. Using an agar-based assay, PLA was the only biopolyester degraded by Savinase, as no activity was observed in the case of PCL, PHB, or PHO over 10 days of incubation at 42 °C (Figure 1F). Unlike Savinase, which is specific toward PLA (Figure 1F), both HiC and cutinase from Cryptococcus sp. are known to degrade other polyesters, like PET, PUR, PCL, and PLA for HiC^32^ and PCL, PHB, and PBS for the cutinase.^33^ In this context, it is conceivable that Savinase could facilitate selective PLA depolymerization in biopolymeric blends and mixed plastic waste. For example, BASF’s ecovio, a blend of PLA and poly­(butylene adipate-*co*-terephthalate) (PBAT), has good potential for stepwise enzymatic depolymerization, as lipase from Pelosinus fermentans DSM 17108 has already been demonstrated to be PBAT specific.^34^ A similar approach has been reported, where Savinase, acting as a broad-specificity protease, achieved over 90% high-purity PET recovery from wool-PET textile blends, enabled by its lack of PET-degrading activity.^35^

Considering that the majority of studies focus on enzyme degradation of powdered PLA substrates with unspecified crystallinity, we aimed to evaluate the performance of Savinase on real-life PLA materials and consumer-grade items (Figure 2).

**Figure 2:**
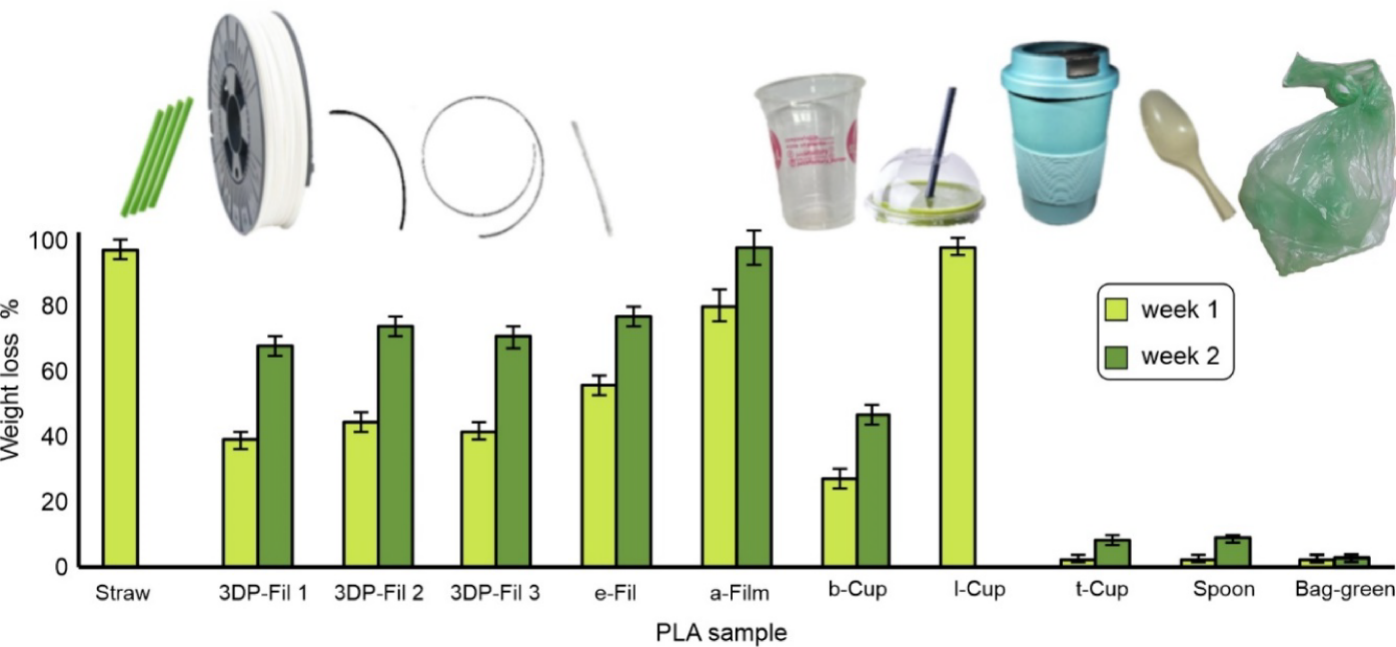
Biocatalytic degradation of commercial grade PLA materials and items by Savinase (0.1% w/w enzyme load) at 42 °C and pH 8.5 during 2 weeks.

All PLA consumer-grade materials were characterized in terms of thermal properties prior to incubation to investigate the influence of crystallinity on their degradation (Table S4). Importantly, all tested PLA materials showed weight loss ranging from 2 wt %, measured for the durable reusable cup (t-Cup) and green bag, to 100 wt % for the single-use cup lid (l-Cup) and PLA straw within the first week, with further degradation occurring during the second week of incubation (Figure 2). Good depolymerization activity was demonstrated on a broad scope of consumer-grade PLA substrates. The l-Cup sample (lid) degraded the fastest, achieving complete degradation within 6 days, followed by the PLA straw that lost 96% of its weight within 7 days (Figure 2). These samples were comparable in thickness, with an *X*_c_ of 22.9% and 11.2%, respectively (Table S4). Therefore, the presence of additives plays a significant role in biodegradation, as both samples showed different thermal and structural properties compared to standard PLA profiles (Figure S2A,C).^36^ Their degradation rates were calculated to be 166 and 137 mg·day^–1^·mg Savinase^–1^, respectively. Proteinase K was reported to achieve comparable degradation of PLA film with unreported crystallinity at a rate of 142 mg·day^–1^·mg Proteinase K^–1^ at 50 °C.^18^ HiC cutinase degraded PLA powder (*X*_c_ 15.2%) at a rate of 30.9 mg·day^–1^·mg HiC^–1^ at 55 °C.^37^ While cutinase from Cryptococcus sp. is reported to be highly efficient at 30 °C, achieving complete PLA powder degradation after 2.5 days with a calculated degradation rate of 200 mg·day^–1^·mg cutinase^–1^, the exact mass of degraded PLA powder was not specified in the study.^33^ Thus, so far, Savinase is the first enzyme efficient in the degradation of consumer-grade PLA materials and items of higher crystallinity at lower temperatures and without pretreatment, including shredding and milling. Other examples of type II PLA depolymerases and their efficiency, usually against powdered materials, are reported in the literature^38–42^ (Table S3). Moreover, the differences observed in enzymatic PLA degradation in this study emphasize the importance of further research on the impact of fillers and additives on enzymatic degradation as a prerequisite for the design of PLA products ready for degradation.^43,44^

The durable reusable cup (t-Cup), the spoon, and the green fruits and vegetables bag were the least susceptible to Savinase degradation under the tested conditions, possibly due to containing additives or having different finishes that inhibited enzymatic adsorption and attack. Indeed, DSC thermograms of both the t-Cup and disposable spoon indicated a shift of the PLA characteristic melting peak to higher values, pointing to blending with other polymers and/or the presence of additives (Table S4, Figures S2A and S3). The spoon also exhibited an additional melting peak at 115 °C (Figure S2A). In comparison, the l-Cup DSC profile showed the characteristic PLA thermal profile that did not change during biodegradation; however, the degree of crystallinity was affected during both hydrolytic and enzymatic degradation (Figure S2B and Table S5). Knowing that PLA is a semicrystalline polymer, containing amorphous and crystalline regions in its conformation, changes in *X*_c_ are a consequence of the increased susceptibility of the amorphous phase to degradation.^45^ Therefore, the *X*_c_ values of the PLA samples increased after degradation due to the preferential degradation of amorphous regions, while the crystalline regions remained stable, resulting in a higher *X*_c_ despite significant weight loss being observed.^17^ Moreover, the reduction of *T*_g_ detected for the l-Cup (Figure 3B) pointed to a reduction in the molecular weight of PLA. As a result of the degradation, the mobility of polymer chains is enhanced due to their scission and the generation of lactic acid oligomers.^46^

**Figure 3:**
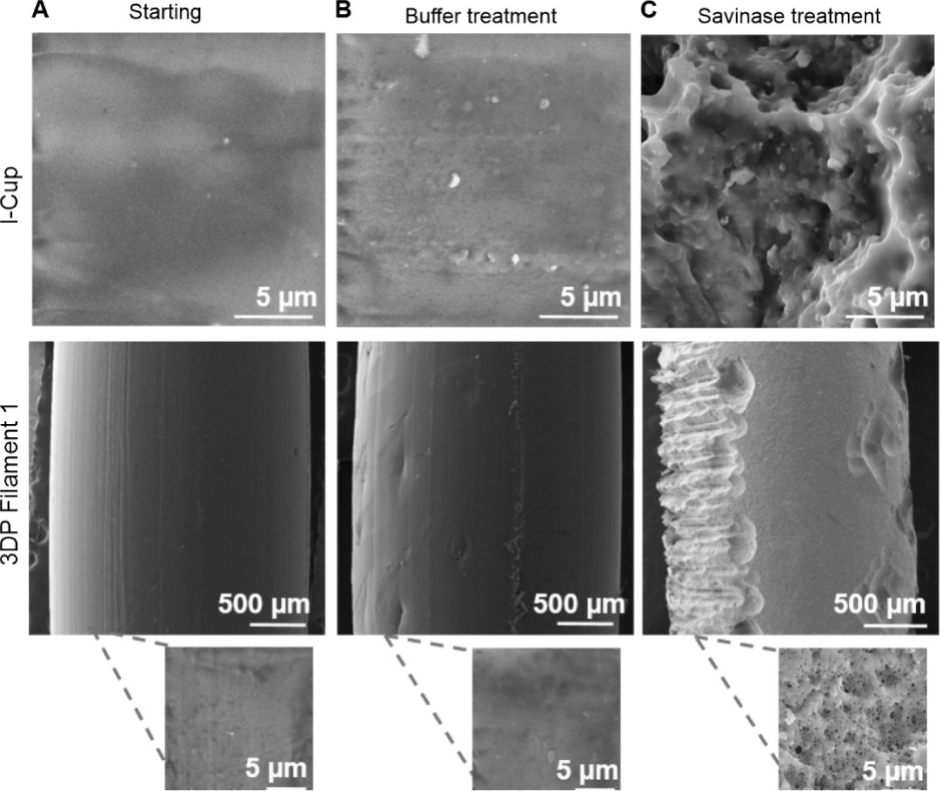
Representative FESEM micrographs of l-Cup and 3D printing filament. (A) Starting materials, (B) incubated in buffer, and (C) degraded by Savinase (magnifications of 80 and 10 k× were used).

Material structural changes during degradation were also monitored using FTIR (Figures S2C,D and S4). The FTIR spectra of all samples except straw and spoon indicated the structure inherent to pure PLA, exhibiting its characteristic peaks.^47^ The strong peak at 1745 cm^–1^ is attributed to −C=O stretching vibrations, while the absorption peaks located at 2886 cm^–1^ are assigned to the −CH_2_ stretching, as well as bands at 1454 cm^–1^ and 1361 cm^–1^ (asymmetric and symmetric −CH_3_ vibrations, respectively).^48^ In the region of 1300 cm^–1^ to 1150 cm^–1^, the C–O stretching modes (ester group) are observed (at 1265 cm^–1^ −C–O bending, at 1182 cm^–1^ (−CO–C– stretching and ν O–C asymmetric mode at 1084 cm^–1^). Finally, in the area of 1000 cm^–1^ and 800 cm^–1^, characteristic peaks observed at 962 cm^–1^ and 843 cm^–1^ can be attributed to the characteristic vibrations of the −C–C– stretching in the PLA amorphous phase.^49^ In addition to DSC evidence, FTIR spectra of the straw and spoon indicated that PLA is probably blended with other polymers or contained additives since the broad peak was detected in the wavenumber region of 800 to 1000 cm^–1^, associated with the additional peak appearing at 1750 cm^–1^, while the main carbonyl peak was shifted to 1715 cm^–1^. Also, in the spectrum of the PLA spoon, a sharp, intense peak at 670 cm^–1^ was also visible. Despite the small weight loss (2.6%) measured for the t-Cup PLA samples, changes in the structure were detected according to the FTIR analysis. Broadening of the characteristic peak located in the area of 800 to 1000 cm^–1^ was observed for the samples tested in the presence of the enzyme (Figure S2D).

Morphological changes in PLA materials upon enzymatic degradation with Savinase were also observed by FESEM (Figure 3). For the representative captured images of PLA samples, the starting sample (Figure 3A) and the sample immersed in buffer (Figure 3B) were presented and compared to samples degraded using Savinase (Figure 3C). The starting PLA consumer-grade samples (l-Cup and 3DP filament) revealed a smooth morphology. Their surface was barely changed after immersion in buffer, with small irregularities and recesses observed, while significant surface erosion and disintegration were detected in enzymatically degraded samples. In general, the surface erosion mechanism through which enzymatic degradation occurs takes place to a greater extent than bulk degradation, resulting in rough and permanent damage to the material surface.^50^ Taken together, the FESEM micrographs provided clear evidence of the biodegradation of all tested PLA consumer-grade samples, showing an increase in porosity and roughness as the degradation progressed.^46^

### Molecular Insights into PLA Degradation by Savinase and Proteinase K

Chromogenic pNP-esters of carboxylic acids are commonly used to determine the monoester-hydrolyzing activity of esterases, including those belonging to the class of type II PLA depolymerases.^51^ So far, there have been individual reports demonstrating Proteinase K and Savinase activity on pNP-acetate and pNP-butyrate, respectively, as type I PLA depolymerases.^52,53^ Therefore, pNP-butyrate was employed to quantify the esterase activity of Savinase and Proteinase K preparations, allowing for the direct comparison of their polyester-hydrolyzing activity (Figure 4).

**Figure 4:**
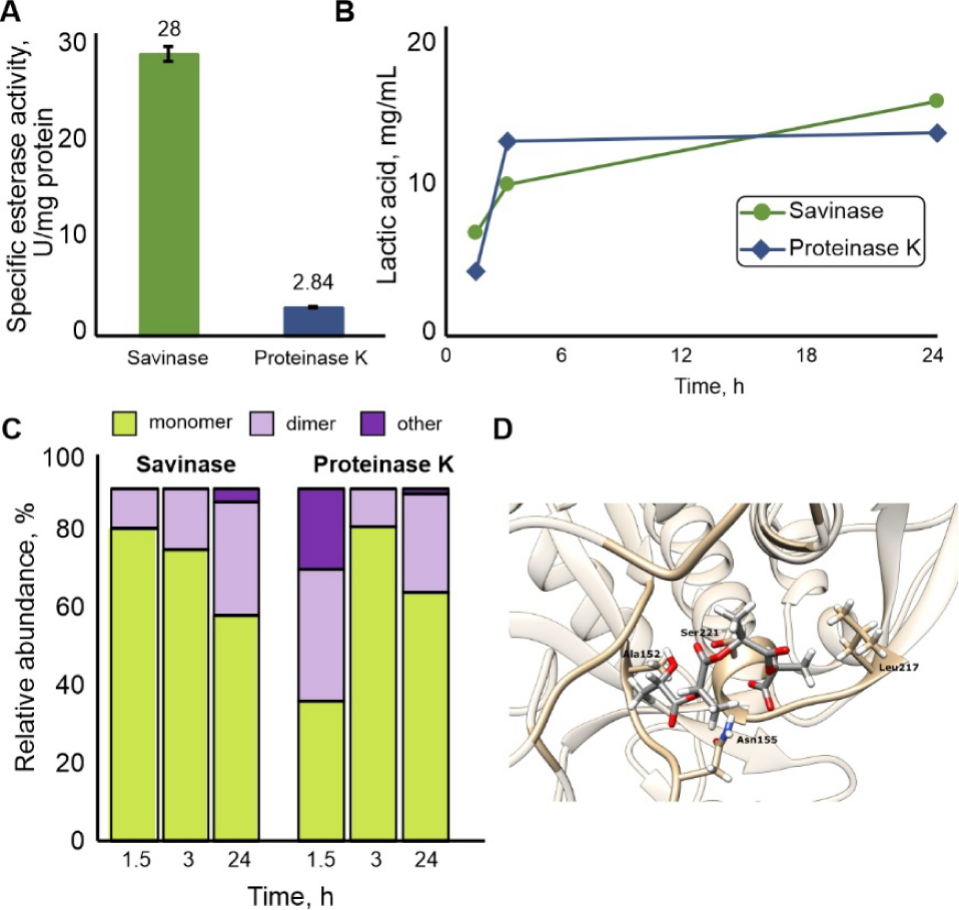
Mode of Savinases’s action on PLA compared to the benchmark Proteinase K and molecular docking of the active site. (A) Monoesterase activity of Savinase and Proteinase K preparations assayed with 0.8 mM pNP-butyrate at pH 8.5 and 25 °C. (B) Lactic acid monomer release during 24 h pure PLA powder degradation by 0.1% w/w Savinase and corresponding esterase units of Proteinase K. (C) Relative abundance of lactic acid to other PLA degradation products. (D) The active site of Savinase in complex with tetrameric L-lactic acid features nucleophilic serine 221 oriented toward the carbonyl of the ester bond between the second and third L-lactic acid residues in the tetramer. Ala152, Asn155, and Leu217 make specific interactions with the tetrameric L-lactic acid within the active site.

Savinase exhibits 10-times higher specific esterase activity toward pNPB than Proteinase K (Figure 4A). Therefore, equal esterase units of 0.1% w/w of Savinase and 1% w/w of Proteinase K were employed in pure PLA powder degradation at 42 °C, pH 8.5, to compare their mode of PLA depolymerization. Lactic acid monomers and dimers were detected as major degradation products in reactions with both enzymes, confirming the effectiveness of the biocatalysts in PLA depolymerization for its recycling (Figure 4C). For the substrates with weight loss higher than 50% (Figure 2), the monomer and dimer detected amounted to between 70 and 90% of the theoretically expected amount, suggesting the presence of other components, such as unidentified additives, in the materials. PLA straw was one of the cleanest samples, with lactic acid release of 3.9 mg·mL^–1^ within 24 h, corresponding to 13.2 mg of PLA converted into calculated 18.6 mg of lactic acid monomer. Savinase and Proteinase K released 6.6 mg·mL^–1^ and 4.1 mg·mL^–1^ of lactic acid after 1.5 h and 15 mg·mL^–1^ and 13 mg·mL^–1^ after 24 h, while exhibiting different lactic acid release kinetics (Figure 4B). The most prominent difference in the hydrolytic profile was observed in the first 1.5 h, where, for Savinase, the lactic acid monomer was present in 2-fold higher relative abundance (89%) compared to other degradation products obtained, whereas only 40% was released by Proteinase K. In addition, within that period, Savinase yielded 11% of PLA dimer, while Proteinase K yielded 37% PLA dimer and 23% of other PLA oligomers (Figure 4C). After 3 h, oligomers were converted by Proteinase K to a comparable ratio of lactic acid monomer (89%) and dimer (11%) as obtained and retained by Savinase in 1.5 and 3 h (Figure 4C). This may be due to the fact that Savinase is retained in the solution by immobilization, favoring the monomerization of PLA degradation products, while Proteinase K binds by adsorption to PLA, preferentially degrading the polymer surface.^45^ During 24 h of PLA depolymerization, the dimer was persistently present, while other oligomers were transient in the case of both enzymes, implying that the dimer degradation rate is somewhat slower than the degradation rates of other oligomers. The appearance of monomeric and oligomeric degradation products suggests both endo- and exo-type PLA scission for Savinase and Proteinase K, consistent with the mode of action of other PLA depolymerases exhibiting both modes of PLA depolymerization.^31^

The molecular docking studies of Savinase with the dimer and tetramer forms of L-lactate using AutoDock Vina, integrated into the SwissDock web server, were performed.^54,55^ The structure of Savinase with PDB ID 1GCI was used for the docking simulations.^56^ The nucleophilic serine residue was selected as the center of the docking box, with a cubic box of dimensions 20 × 20 × 20 Å defined around it to encompass the active site and ensure precise ligand docking. This approach allowed us to explore the binding interactions and affinities of the lactate oligomers within enzyme’s active site. Our results indicated that Savinase exhibited binding affinities of −4.710 kcal/mol for the tetramer of L-lactic acid and −4.190 kcal/mol for the dimer of L-lactic acid, suggesting Savinase preferably hydrolyzes longer-chain PLA fragments. The 20 highest-ranking poses are provided in Table S6, and the pose with the highest affinity is illustrated in Figure 4D. In Savinase, Asn155 forms a favorable interaction with the carboxylate group of the L-lactic acid tetramer, while the carbonyl group of a peptide bond in Ser125 interacts with the hydroxyl (OH) group at the OH end of the L-lactic acid tetramer. The terminal methyl groups at both ends of the tetrameric L-lactic acid interact with the side chains of Ala152 and Leu217. For the dimer of L-lactic acid, although high binding affinities were observed, most predicted poses were nonproductive. However, one pose with an affinity of −4.033 kcal/mol showed potential, with the nucleophilic oxygen of Ser221 positioned 3.13 Å from the carbonyl carbon of the dimer. To further explore the binding of the L-lactic acid dimer, molecular dynamics simulations were conducted. In all three replicas, the dimer departed from the active site and moved into the bulk water, consistent with the observation that lactic acid dimers were detected as significant degradation products in reactions with both enzymes (Figure 4C). In contrast, PCL, PHB, and PHO dimers resulted in nonproductive binding poses: there was no close contact or favorable orientation between the nucleophilic serine residue (Ser221) of Savinase and the carbonyl carbon of the ester bond in any of the substrates (Figure S5). This lack of catalytic alignment supported the experimental observation that Savinase was inactive against PCL, PHB, and PHO (Figure 1F). The reason for this contrast between the biopolymers lies in their structural differences. PLA consists of repeating L-lactate units linked by ester bonds, which mimic the geometry of peptide bonds found in natural proteins composed of L-amino acids.^31^ PCL has longer aliphatic chains (six carbon atoms between ester groups, versus three in PLA), which introduce steric hindrance and flexibility that reduce substrate fit within the protease’s active site. PHB and PHO are composed of 3-hydroxyalkanoate monomers with bulkier side chains, which do not complement the shape and polarity of Savinase’s active site. Thus, Savinase’s selectivity for PLA arises from its evolutionary adaptation to peptide bond hydrolysis, with PLA’s stereochemistry and ester bond orientation closely resembling natural substrates of proteases.

Inspired by self-degrading PLA materials developed by Guicherd et al., obtained by embedding engineered protease into PLA,^57^ we incorporated Savinase into PLA filaments for 3D printing to further broaden Savinase application. The thermal characteristics of PLA filaments with embedded Savinase are listed in Table S7. Embedding Savinase into PLA did not yield self-degrading PLA material, as confirmed upon incubation in buffer (Figure S6), suggesting thermal inactivation of the enzyme during the high-temperature processing necessary for PLA (160 °C) (Table S2). Similar results were obtained with the melt extrusion of ProteinT^FLTIER^ into PLA, in both liquid and solid forms, even when enzyme’s thermal stability is increased by protein engineering (Tm 79.4 °C)^57^. Therefore, further optimization of the melt extrusion process is necessary, including enzyme precoating in PCL or polyacrylamide at low temperatures.^57,58^

Nevertheless, the applicability of Savinase in alternative recycling strategies could be further expanded by simplifying the approach and using it as a composting enhancer. After six months of incubation, consumer-grade PLA bags from both the control and the Savinase-supplemented compost exhibited significant degradation, with Savinase treatment being 2-fold more efficient (Figure S7). Notable visual changes included alterations in color and the fragmentation of the bags, with the remaining pieces in the supplemented compost being visibly smaller and more fragile, suggesting that Savinase could be used as a composting enhancer. A lower temperature optimum and broad temperature range of Savinase, as well as a cost-effective production route, make it suitable for this application.

Overall, the enzymatic PLA degradation is a scientifically mature but industrially emerging field; therefore, the novel contribution of this study lies in the comprehensive characterization of the depolymerization of consumer-grade PLA by Savinase, a commercially available and industrially scalable enzyme. Savinase’s polymeric substrate selectivity, its optimal PLA depolymerizing conditions, and its PLA depolymerization mode of activity compared to Proteinase K are reported for the first time. Savinase demonstrated specificity toward PLA among other bioplastics, which contributes new insights to the field of polymer-selective enzymes, important for effective mixed-plastic depolymerization. Identification of key active-site residues responsible for specific PLA recognition by molecular dynamics simulation and the kinetics of product release filled a research gap in the mechanistic understanding of PLA depolymerases, as highlighted by the recent systematic review.^10^ Up until now, the mode of enzymatic PLA depolymerization was addressed only for type II PLA depolymerases (MGS0156, GEN0105, ABO2449, RPA1511, and PaE),^31^ while this study provided novel insights into the mode of action of type I PLA depolymerases. Furthermore, this study demonstrated that less-studied immobilized enzymes^57–60^ can achieve high PLA degradation rates, with added benefits of reusability and simplified product recovery. Future efforts should be aimed at the discovery and engineering of PLA-degrading enzymes that work at moderate temperatures (30 °C) and ambient pH and can tolerate a wide range of additives. Efforts to design bioreactors for PLA waste treatment should also be made, as these could contribute to efficient integration into the circular economy of plastics. Enzymatic hydrolysis of PLA produces lactic acid, which can be recovered and repurposed for the synthesis of new PLA or even other microbial biopolymers (i.e., PHAs or nanocellulose).

## Conclusions

Savinase showed high specificity toward PLA in comparison to other bioplastics, with Asn155 and Ser125 identified as key residues involved in PLA recognition. Savinase is a suitable catalyst for PLA depolymerization in mixed plastic waste, as it can eliminate the necessity for plastic waste sorting. Endo- and exo-type PLA scission mechanisms were revealed, with lactic acid monomer released as the main degradation product, showcasing its potential for application in PLA monomer recycling. PLA degradation by Savinase occurs at reduced temperatures in comparison to industrial composting (∼60 °C) and benchmark Proteinase K (50 °C). Therefore, Savinase can contribute to energy savings during large-scale PLA depolymerization and can be used as a home-composting enhancer. Unlike other PLA depolymerases, Savinase is an enzyme with an already established large-scale production route and, as such, is currently available on the market. Savinase versatility in PLA degradation at mesophilic temperatures is demonstrated across a wide range of mass-produced PLA substrates. Variations in PLA degradation for different consumer-grade products in this study further emphasized the need for more extensive investigations into the influence of fillers/additives on enzymatic degradation to produce designed-to-degrade PLA items.

## Supplementary Material



## Notes

Additional experimental details for instrumental analysis and embedding Savinase into PLA; FTIR and LC-MS spectra; PLA thermograms; docking of PCL, PHB, and PHO dimers; images of degradation of PLA with embedded Savinase and PLA composting (PDF)
