# Supplementary material for: Efficient Degradation of Consumer-Grade PLA by Commercial Savinase: Optimized Conditions and Molecular Dynamics Insights
Source: ACS Sustain Chem Eng. 2025 Jun 9;13(24):9269–78. doi: 10.1021/acssuschemeng.5c03378 (PMC12188634; doi:10.1021/acssuschemeng.5c03378)
Supplement: Supplementary file 1 [file sc5c03378_si_001.pdf]

SUPPORTING INFORMATION

Efficient Degradation of Consumer-Grade PLA by Commercial Savinase: Optimized Conditions and Molecular Dynamics Insights

Marija Nenadović[†], Marijana Ponjavić[†], Brana Pantelic[†], Maciej Guzik[‡], Tomasz M. Majka[§], Georgia Sourkouni[‖], Aleksandra Maršavelski[#], Jasmina Nikodinovic Runic*[†]

[†] *Institute of Molecular Genetics and Genetic Engineering, University of Belgrade, Vojvode Stepe 444a, Belgrade, 11000, Serbia*

[‡] *Jerzy Haber Institute of Catalysis and Surface Chemistry, Polish Academy of Sciences, Niezapominajek 8., Krakow, 30-239, Poland*

[§] *Department of Chemistry and Technology of Polymers, Faculty of Chemical Engineering and Technology, Cracow University of Technology, Warszawska 24, Krakow, 31-155, Poland*

[‖] *Clausthal Centre for Materials Technology (CZM), Clausthal University of Technology, Leibnizstraße 9, Clausthal-Zellerfeld, 38678, Germany*

[#] *Faculty of Science, University of Zagreb,* Horvatovac 102a*, Zagreb, 10000, Croatia*

*Email: jasmina.nikodinovic@imgge.bg.ac.rs; Phone:00 381 11 397 60 34

Number of pages: 19

Number of figures: 5

Number of tables: 7

Contents

Additional experimental details………………………………………………… S3

Figure S1. LC-MS identification of lactic acid monomer and dimer during enzymatic PLA with Savinase and Proteinase K ……….. S5

Figure S2. Thermal and surface assessment of representative consumer-grade PLA materials…………………………………………………………………………… S6

Figure S3. DSC curves of the t-cup PLA consumer grade before and after degradation S7

Figure S4. FTIR analysis of the starting PLA consumer grade samples…………… S8

Figure S5. The active site of Savinase in complex with the dimers of A) 6-hydroxy hexanoic acid (representing PCL), (B) 3-hydroxybutyric acid (representing PHB), and (C) 3-hydroxyoctanoic acid (representing PHO), biopolyesters that were not hydrolyzed. Each compound is shown in blue, and the catalytic triad is depicted as tan-colored sticks………………………………………………………………………… S9

Figure S6. Biodegradation of PLA with varying amounts of incorporated Savinase S10

Figure S7. PLA soil-composting with Savinase…………………………………… S11

Table S1. Properties of commercial grade PLA used in this study………………… S12

Table S2. e-Filament with embedded Savinase processing conditions…………….. S13

Table S3. Comparison of Savinase and other PLA degrading enzymes…………… S14

Table S4. DSC analysis results of consumer-grade PLA materials………………… S15

Table S5. DSC analysis results of selected PLA samples before and after degradation S16

Table S6. The docking simulations to estimate binding of dimer and tetramer of L-lactic acid……………………………………………………………………………… S17

Table S7. Thermal characteristics of PLA filaments with embedded Savinase…… S18

References………………………………………………………………………. S19

Additional experimental details

Embedding of Savinase into PLA materials.

In the preparation e-Filament process, a high-temperature processing line consisting of a Brabender DR20 feeder (RHL-Service, Poznan, Poland), twin screw extruder Thermo Scientific Haake Rheomex OS PTW 16/25 (RHL-Service, Poznan, Poland) was used. PLA and PLA/Savinase 12T composites containing 0.5, 1.0 and 1.5 wt % of enzyme were obtained using this processing line. The processing conditions are shown in Table S2. Samples with embedded enzyme (approx. 100 mg) were incubated in water to assess biodegradation. For the degradation under aqueous conditions, PLA samples were cut to 50 mg and incubated in 10 mL of 0.1 M Tris buffer (pH 8.5) for 2 weeks at 42 °C with shaking at 220 rpm. Additionally, set of reaction contained 0.05% w/w Savinase in the same reaction buffer. After degradation, all samples were rinsed with distilled water and 70% (V/V) ethanol, dried, and weighed and weight loss was calculated.

PLA samples preparation and characterization.

Confocal Laser Scanning Microscopy (CLSM). CLSM was used for hc-Films surface visualization before and after degradation by Savinase. After degradation, the PLA film was rinsed three times with water and 70% ethanol, and dried. The measurements were conducted utilizing the VK-X200K microscope from KEYENCE using the lateral resolution of 160 nm. The recorded topography of the sample enables performing roughness analyses or profile sections [1].

Fourier Transform Infrared Spectroscopy (FTIR) analysis. The FTIR spectra of the PLA samples before and after degradation were acquired using an IR-Affinity spectrometer (Thermo Fisher Scientific, NICOLET iS10, Waltham, MA, USA) in attenuated total reflection (ATR) mode. Measurements were conducted in the wavenumber range of 4000 to 400 cm^{-1}, at room temperature, with a resolution of 4 cm^{-1} and with the fixed number of scans at 32.

Differential Scanning Calorimetry (DSC) analysis. Differential Scanning Calorimetry (DSC) measurements were performed using a Shimadzu– DSC-60 Plus in the nitrogen atmosphere, gas flow of 50 $mL \cdot min^{-1}$. Indium was used to calibrate the calorimeter in temperature and energy. All the measurements were performed in the temperature range from -50 to 250 °C, at a heating rate of 10 °C min^{-1}. Sample weight was approximately 5.0 ± 0.5 mg. The determination of melting temperature, T_m, glas transition temperature, T_g, temperature of crystallization, T_c, as well as the

coresponding enthalpies, (ΔH_m, ΔH_c) obtained from DSC, was done using TRIOS Software TA Universal Data Analysis. The obtained melting enthalpies were determined from the area under the endotherms, while the degree of crystallinity, X_c, was defined from the calculated melting enthalpie, ΔH_m, and the melting enthalpy of 100% crystalline PLA, ΔH_m°, (93.6 J g^{-1}) [2] following the eqn (1):

$$X_c = \frac{\Delta H_m}{\Delta H_m^{\circ}} \times 100\ \% \quad (1).$$

Field Emission Scanning Electron Microscopy (FESEM) analysis. The morphology of the investigated PLA materials before and after degradation was analyzed using FESEM. Surface modifications, including those referring the degradation, were explored using the FESEM Mira3 Tescan model at an accelerating voltage of 10 kV. Prior to imaging, samples were attached on double-sided carbon adhesive tape on aluminum studs and sputter-coated with a thin layer of Au.

HPLC and LC-MS run conditions.

The isocratic HPLC separation on HypersylGold C18 (150 × 4.6 mm) column (Thermo Scientific, USA) was run at a flow rate of 1 mL·min^{-1}, with the mobile phase consisting of 95% (v/v) 20 mM $NH_4H_2PO_4$ in ultrapure water (pH adjusted to 2.0 with H_3PO_4) and 5% (v/v) acetonitrile for 30 min per sample. The degradation products were detected at λ = 210 nm.

For LC-MS sample analysis TSQ Fortis Plus Triple Quadrupole Mass Spectrometer (Thermo Scientific, USA) with an H-ESI source was used in mixed scan mode and single ion monitoring (SIM) scan type. The isocratic separation on Acclaim Polar Advantage II C18 (2.1 x 50 mm) column (Thermo Scientific, USA) was run with a mobile phase composed of 50% (v/v) acetonitrile and 50% (v/v) Milli-Q water with 0.1% formic acid for 18 min per sample. Ionization parameters were as follows: a positive spray voltage of 4300 V, a negative spray voltage of 3500 V, a sheath gas flow rate of 12 arbitrary units (arb), an auxiliary gas flow rate of 8 arb, a sweep gas flow rate of 1 arb, an ion transfer tube temperature of 300 °C, and a vaporizer temperature of 350 °C.

Supporting Figures

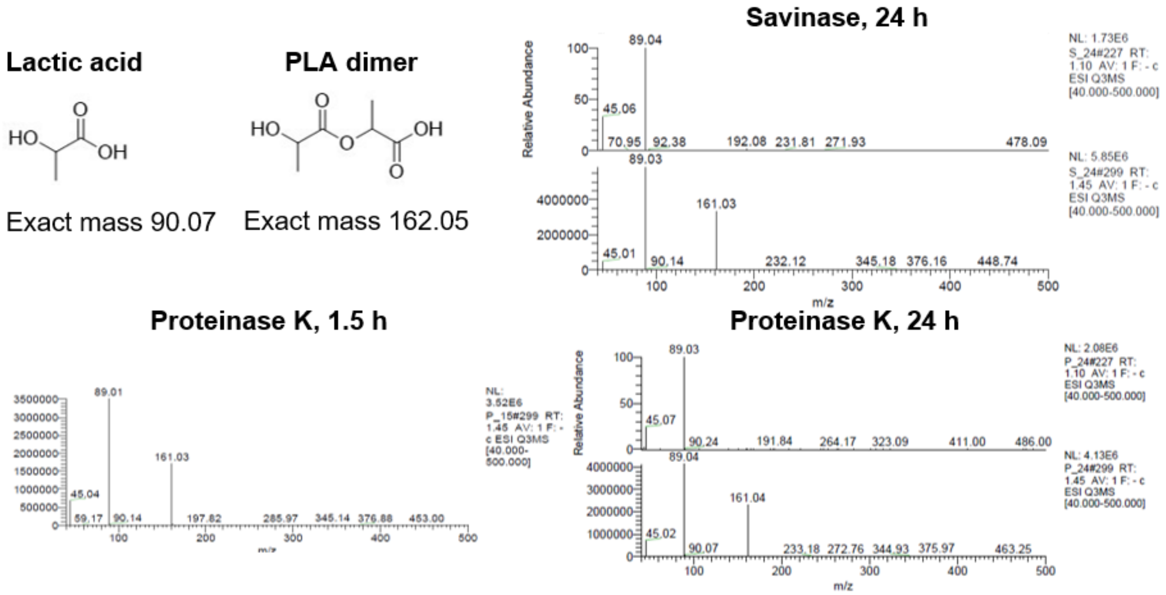

Figure S1. LC-MS identification of lactic acid monomer and dimer released during 24 h enzymatic hydrolysis of pure PLA powder with Savinase and Proteinase K.

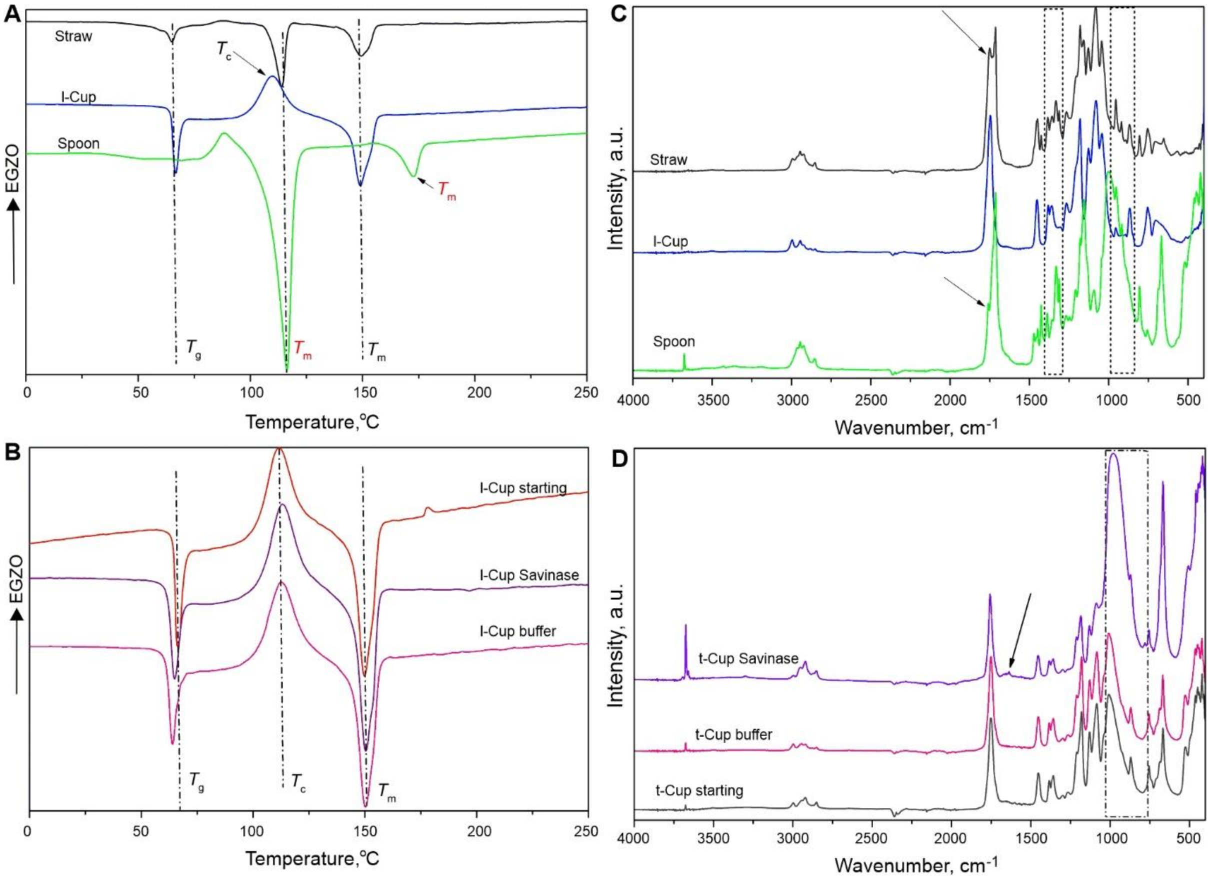

Figure S2. Thermal and surface assessment of representative consumer-grade PLA materials. A) DSC analysis of straw, l-Cup and spoon starting samples, B) DSC analysis of l-Cup samples before and after degradation experiment in buffer and with the added Savinase, C) FTIR analysis of starting straw, l-cup and spoon consumer-grade samples (changes in the characteristic peaks are marked with arrows), and D) FTIR analysis of t-Cup samples before and after degradation (different appearance of the characteristic peak marked with arrow).

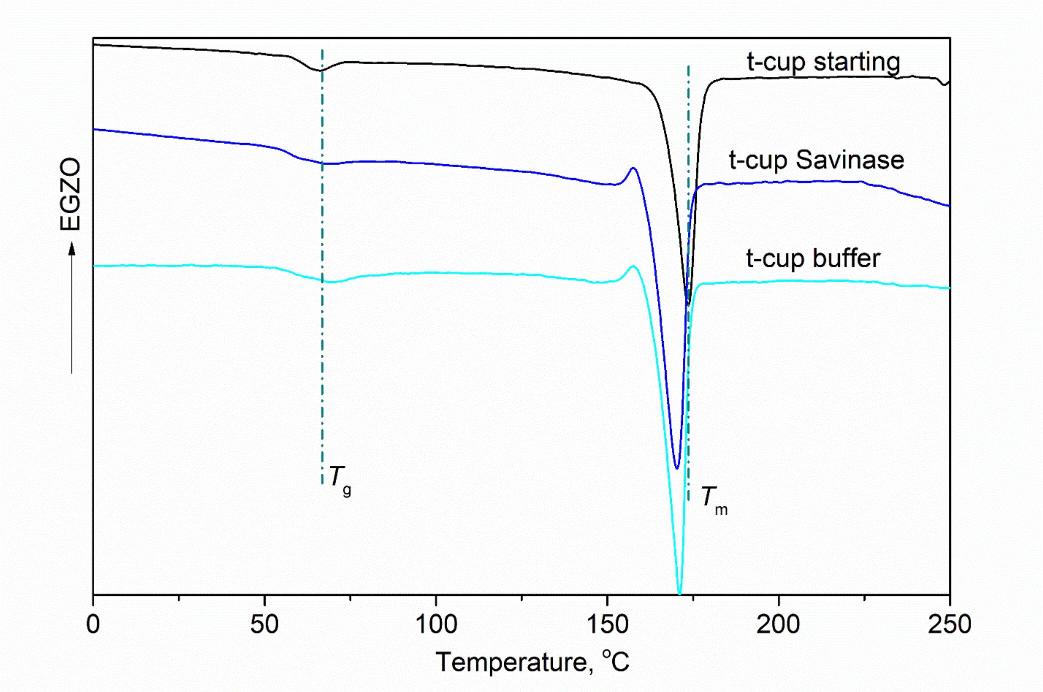

Figure S3. DSC curves of the t-cup PLA consumer grade before and after degradation.

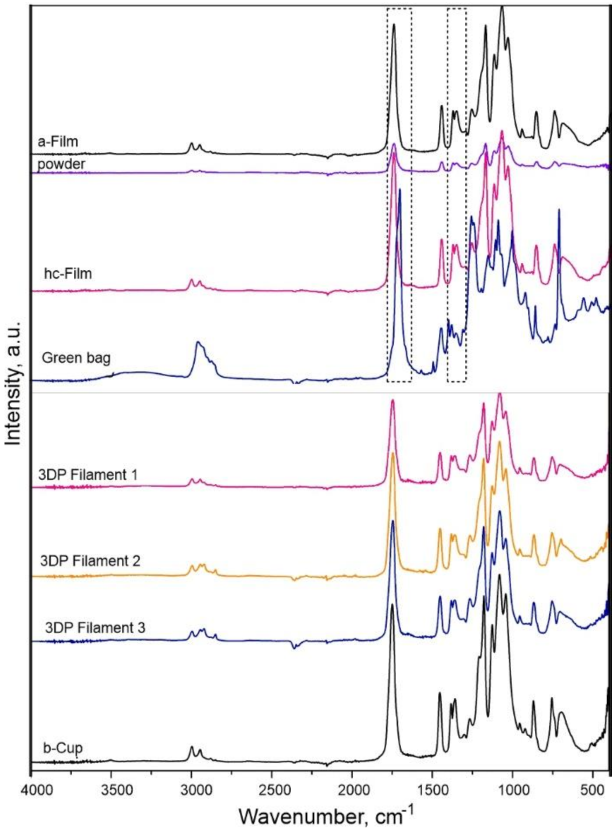

Figure S4. FTIR analysis of the starting PLA consumer grade samples.

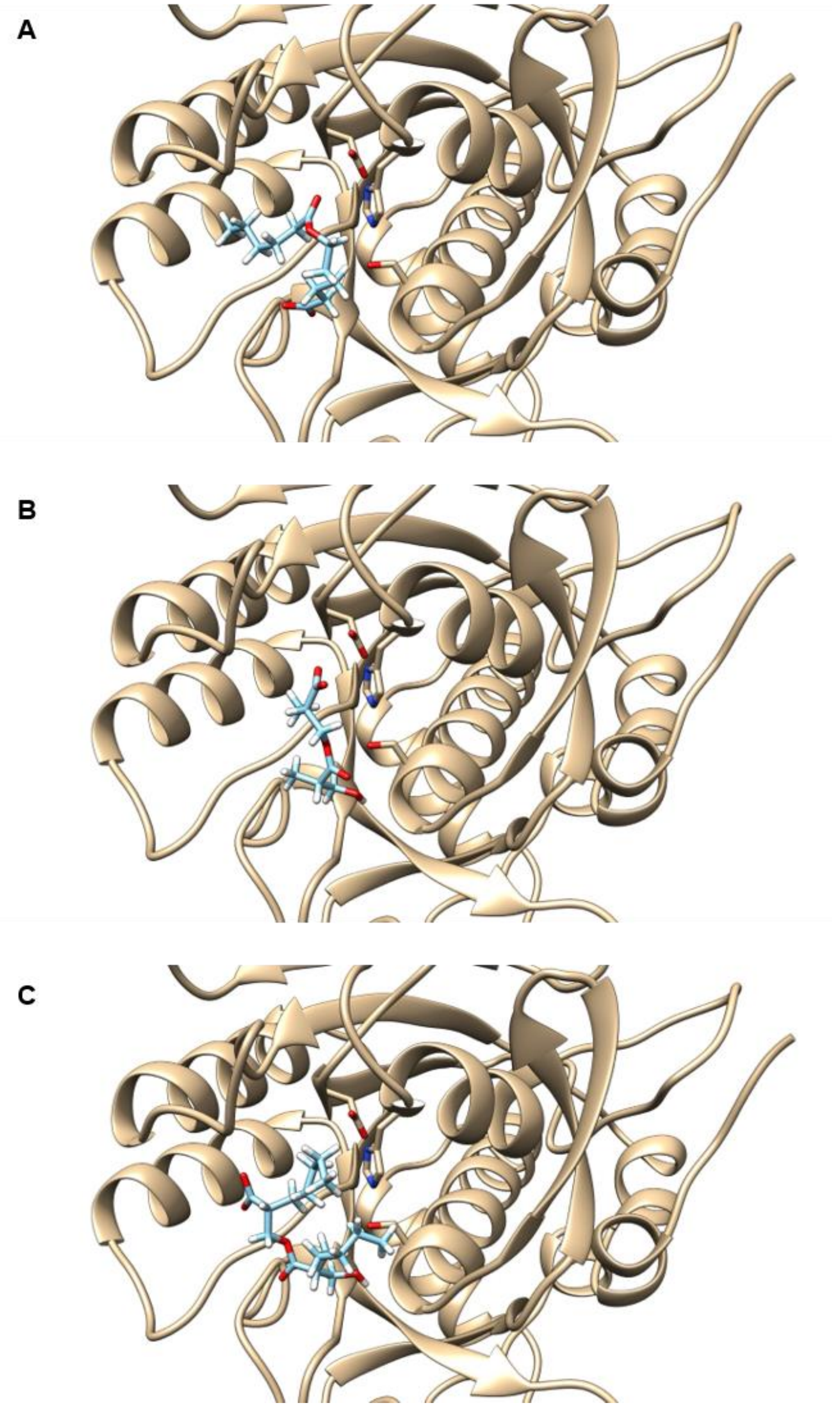

Figure S5. The active site of Savinase in complex with the dimers of A) 6-hydroxyhexanoic acid (representing PCL), (B) 3-hydroxybutyric acid (representing PHB), and (C) 3-hydroxyoctanoic acid (representing PHO), biopolyesters that were not hydrolyzed. Each compound is shown in blue, and the catalytic triad is depicted as tan-colored sticks.

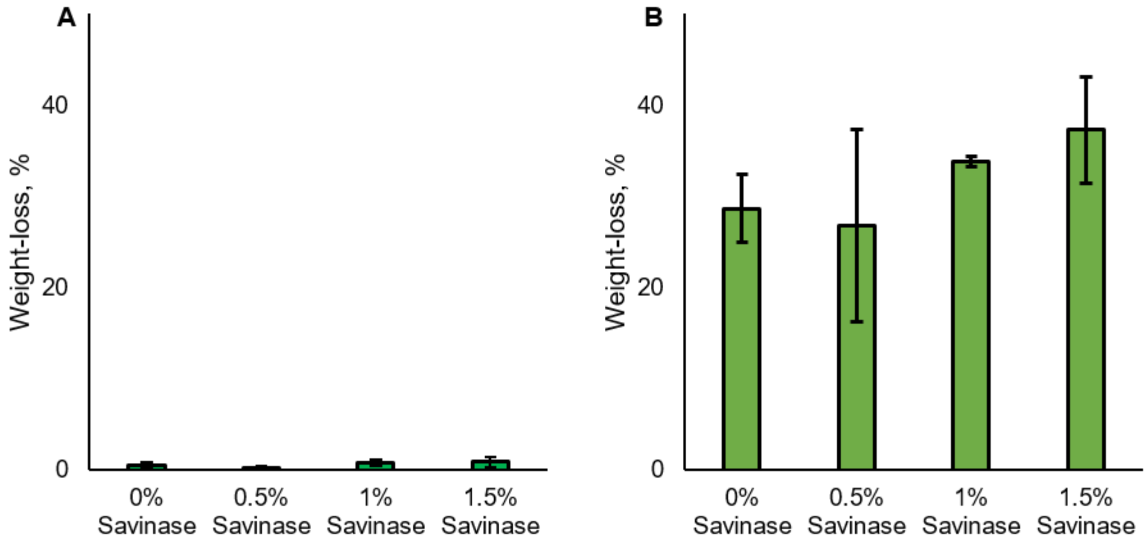

Figure S6. Biodegradation of PLA with varying amounts of incorporated Savinase at 42 °C, pH 8.5 in the A) buffer and B) the buffer containing 0.05% Savinase after 2 weeks.

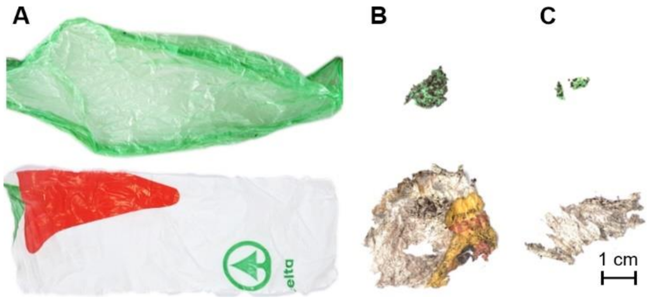

Figure S7. PLA soil-composting with Savinase A) PLA bags before soil burial; B) PLA bags fragments after six months of incubation in control compost and C) compost supplemented with Savinase.

Table S1. Properties of commercial grade PLA used in this study

Sample	Supplier	Dimensions (L x H)	Thickness (mm)	Initial mass (mg)
Straw	KingPack (https://kingpack.co.za)	1 cm × 6 mm	0.16	33
3DP-Filament 1	3D republika (https://3drepublika.com/)	1 cm × 2.85 mm	2.85	80
3DP-Filament 2	3D republika (https://3drepublika.com/)	1 cm × 2.95 mm	2.95	82
3DP-Filament 3	3D republika (https://3drepublika.com/)	1 cm × 1.75 mm	1.75	30
e-Filament	NatureWorks (PLA Ingeo 3052D)	1 cm × 1.2 mm	1.2	11
a-Film	NatureWorks (PLA Ingeo 4043D)	1 cm × 1 cm	0.35	52
b-Cup	JuiceFactory (https://www.juicefactory.at/)	1 cm × 1 cm	0.2	27
l-Cup	JuiceFactory (https://www.juicefactory.at/)	1 cm × 1 cm	0.18	25
t-Cup	CHIC.MIC (https://haendler.chicmic.de/)	1 cm × 1 cm	2.45	134
Spoon	BioTable (https://biotable.it/)	1 cm × 1 cm	0.6	83
Powder	NatureWorks (PLA Ingeo 4043D)	<500 µm[a]		33
hc-Film	Pardam nanotechnology (https://www.pardam.cz/)	1 cm ×1 cm	1	141
Bag-green	Lidl fruits and vegetables packaging bag	4 cm × 4 cm	0.01	23

[a] Average particle size

Table S2. e-Filament with embedded Savinase processing conditions

Extruder							
	**Heating zones**						
**Temperature (°C)**	**1**	**2**	**3**	**4**	**5**	**6**	**Die**
	180	180	185	190	195	200	200
**Degassing**	-	-	-	-	Yes	-	-
**Screws speed (rpm)**	150						
**Feed capacity (%)**	5						

Table S3. Comparison of Savinase and other PLA degrading enzymes

Enzyme	Microbial origin	Plastic substrates	Used PLA substrate	Reaction conditions	Enzynme amount	Optimum conditions	Degradation efficiency	Comparison to Proteinase K	Ref.
PLA depolymerase I (EC 3.4.-.-, protease)									
Savinase	*Bacillus lentus*	PLA	PLA film:<br>-Xc 16.5<br><br>PLA straw<br>-Xc 11.2 %<br><br>PLA single-use cup<br>-Lid - Xc 22.9 %<br>-Bottom - Xc 42.0 %<br><br>PLA 3D printer filamets:<br>-Xc 25.6 %<br>-Xc 20.7 %<br>- Xc 22.2 %	42°C, pH 8.5	0.1% (w/w)	42°C, pH 8.5	PLA films:<br>-116 mg·day^{-1}·mg enzyme^{-1}<br><br>Straw<br>- 137 mg·day^{-1}·mg enzyme^{-1}<br>Single use cup<br>-166 mg·day^{-1}·mg enzyme^{-1}<br>- 40 mg·day^{-1}·mg enzyme^{-1}<br><br>3D printer filament<br>-56 mg·day^{-1}·mg enzyme^{-1}<br>-65 mg·day^{-1}·mg enzyme^{-1}<br>-60 mg·day^{-1}·mg enzyme^{-1}	Same mode of PLA depolymerization	
Proteinase K	*Tritirachium album*	PLLA[a]	PLA film (12 mg)	50°C, pH 8.6	/	50°C[b] pH 8.5	142 mg·day^{-1}·mg enzyme^{-1}	/	[3] [a4] [b5]
Protease T16-1	*Actinomadura keratinolytica* T16-1	PLA	PLA powder (20 mg)	60°C, pH 9	600 U	70°C, pH 10[b]	16.4 mg·day^{-1}	Equal activity	[6] [b7]
PLA depolymerase II (EC 3.1.1.-, carboxylic-ester hydrolases)									
HiC	*Humicola insolens*	PLLA, PDLA, PDLLA, PURb, PETa	PLA powder (Xc 24%, 150 mg)	55°C, pH 9	0.65% (w/w)	55°C, pH 8	30.9 mg·day^{-1}·mg enzyme^{-1}	/	[8]
Cutinase CS2	*Cryptococcus* sp. Strain S-2	PLA, PBS, PCL, PHB	PLA powder (0.04%)	30°C, pH 8	0.2-25%	/	200 mg·day^{-1}·mg enzyme^{-1}	550x higher activity	[9]

Table S4. DSC analysis results of consumer-grade PLA materials tested in this study

Sample	T_m, °C[a]	ΔH_m, J/g[b]	X_c, %[c]	T_g, °C[d]	T_c, °C[e]	ΔH_c, J/g[f]
Straw	149.3	10.80	11.2	65.1	ND	ND
3DP-Filament 1	155.1	24.0	25.6	66.6	104.1	27.1
3DP-Filament 2	150.0	19.40	20.7	66.3	109.6	19.8
3DP-Filament 3	151.3	20.8	22.2	67.0	114.0	20.0
e-Filament	150.7	27.4	29.3	64.4	116.3	30.0
a-Film	153.9	6.8	7.30	66.0	119.8	8.50
b-Cup	149.6	39.3	42.0	72.4	ND	ND
l-Cup	150.0	21.4	22.9	66.6	109.8	20.22
t-Cup	173.3	22.0	23.6	65.7	ND	ND
Spoon	155.8	1.60	1.70	68.7	116.3	61.7
Powder	155.1	14.2	15.2	67.9	121.8	14.1
hc-Film	153.9	27.7	29.6	66.2	115.8	16.2
Green-bag	156.4	1.20	1.28	60.3	ND	ND

[a] Melting temperature, T_m; [b] Melting enthalpy, ΔH_m

[c] X_c value was calculated according to the equation (1) without including the exact amount of PLA (w_{PLA}) which is known to the manufacturer and it is not stated in the product declaration.

[d] Glass transition temperature, T_g; [e] Crystallization temperature, T_c; [f] Crystallization enthalpy, ΔH_c

Table S5. DSC analysis results of selected PLA samples before and after degradation study

Sample	T_m, °C	ΔH_m, J/g	X_c, %
l-Cup starting	149.8	18.6	19.9
l-Cup buffer	150.0	17.5	18.7
l-Cup Savinase	150.4	18.4	19.7
t-Cup starting	173.3	22.0	23.6
t-Cup buffer	171.2	29.3	31.3
t-Cup Savinase	170.4	29.9	31.9
hc-Film starting	153.9	27.7	29.6
hc-Film buffer	158.9	39.6	42.3
hc-Film Savinase	158.8	31.5	33.7

Table S6. The docking simulations to estimate binding of dimer and tetramer of L-lactic acids were performed using AutoDock Vina, integrated into the SwissDock web server.

**Model**<br>**Savinase (PDB ID 1GCI) + dimer of L-lactic acid**	**ΔG,**<br>**kcal/mol**
1	-4.190
2	-4.093
3	-4.055
4	-4.033
5	-4.013
6	-3.961
7	-3.930
8	-3.858
9	-3.830
10	-3.828
11	-3.674
12	-3.673
13	-3.617
14	-3.603
15	-3.560
16	-3.535
17	-3.513
18	-3.502
19	-3.493
20	-3.478

**Model**<br>**Savinase (PDB ID 1GCI) + tetramer of L-lactic acid**	**ΔG,**<br>**kcal/mol**
1	-4.710
2	-4.689
3	-4.608
4	-4.584
5	-4.497
6	-4.462
7	-4.460
8	-4.459
9	-4.435
10	-4.412
11	-4.410
12	-4.402
13	-4.400
14	-4.392
15	-4.368
16	-4.353
17	-4.297
18	-4.251
19	-4.215
20	-3.704

Table S7. Thermal characteristics of PLA filaments with embedded Savinase

Sample/%Enzyme	T_m, ºC	ΔH_m, J/g	X_c, %	T_g, ºC	T_c, ºC	ΔH_c, J/g
PLA control	150.7	27.4	61.7	64.4	116.3	30.0
PLA 0.5	152.3	13.5	30.1	66.2	117.0	14.4
PLA 1.0	151.6	11.8	26.0	64.6	120.6	12.2
PLA 1.5	152.5	12.5	28.1	64.7	121.6	13.2

REFERENCES

(1) Sourkouni, G.; Kalogirou, C.; Moritz, P.; Gödde, A.; Pandis, P. K.; Höfft, O.; Vouyiouka, S.; Zorpas, A. A.; Argirusis, C. Study on the Influence of Advanced Treatment Processes on the Surface Properties of Polylactic Acid for a Bio-Based Circular Economy for Plastics. *Ultrason. Sonochem.* **2021**, *76*, 105627. https://doi.org/10.1016/j.ultsonch.2021.105627.

(2) Katzenberg, F.; Tiller, J. C. Shape Memory Natural Rubber. *J. Polym. Sci. Part B Polym. Phys.* **2016**, *54* (14), 1381–1388. https://doi.org/10.1002/polb.24040.

(3) Oda, Y.; Yonetsu, A.; Urakami, T.; Tonomura, K. Degradation of Polylactide by Commercial Proteases. *J. Polym. Environ.* **2000**, *8*, 29–32. https://doi.org/10.1023/A:1010120128048.

(4) Reeve, M. S.; McCarthy, S. P.; Downey, M. J.; Gross, R. A. Polylactide Stereochemistry: Effect on Enzymic Degradability. *Macromolecules* **1994**, *27* (3), 825–831. https://doi.org/10.1021/ma00081a030.

(5) Cui, L.; Wang, X.; Szarka, G.; Hegyesi, N.; Wang, Y.; Sui, X.; Pukánszky, B. Quantitative Analysis of Factors Determining the Enzymatic Degradation of Poly(Lactic Acid). *Int. J. Biol. Macromol.* **2022**, *209*, 1703–1709. https://doi.org/10.1016/j.ijbiomac.2022.04.121.

(6) Youngpreda, A.; Panyachanakul, T.; Kitpreechavanich, V.; Sirisansaneeyakul, S.; Suksamrarn, S.; Tokuyama, S.; Krajangsang, S. Optimization of Poly(Dl-Lactic Acid) Degradation and Evaluation of Biological Re-Polymerization. *J. Polym. Environ.* **2017**, *25* (4), 1131–1139. https://doi.org/10.1007/s10924-016-0885-1.

(7) Sukkhum, S.; Tokuyama, S.; Tamura, T.; Kitpreechavanich, V. A Novel Poly (L-Lactide) Degrading Actinomycetes Isolated from Thai Forest Soil, Phylogenic Relationship and the Enzyme Characterization. *J. Gen. Appl. Microbiol.* **2009**, *55* (6), 459–467. https://doi.org/10.2323/jgam.55.459.

(8) Pérez-Venegas, M.; Friščić, T.; Auclair, K. Efficient Mechano-Enzymatic Hydrolysis of Polylactic Acid under Moist-Solid Conditions. *ACS Sustain. Chem. Eng.* **2023**, *11* (27), 9924–9931. https://doi.org/10.1021/acssuschemeng.2c06847.

(9) Masaki, K.; Kamini, N. R.; Ikeda, H.; Iefuji, H. Cutinase-Like Enzyme from the Yeast Cryptococcus Sp. Strain S-2 Hydrolyzes Polylactic Acid and Other Biodegradable Plastics. *Appl. Environ. Microbiol.* **2005**, *71* (11), 7548–7550. https://doi.org/10.1128/AEM.71.11.7548-7550.2005.
